# Attitudes towards embryo donation among healthcare professionals working in child healthcare: a survey study

**DOI:** 10.1186/s12887-019-1578-4

**Published:** 2019-06-25

**Authors:** Gabriela Armuand, Gunilla Sydsjö, Agneta Skoog Svanberg, Claudia Lampic

**Affiliations:** 1 Department of Clinical and Experimental Medicine, Faculty of Health Sciences, Linköping University, SE-581 83 Linköping, Sweden; 2 Department of Gynaecology and Obstetrics in Linköping, County Council of Östergötland, Linköping, Sweden; 3 Department of Women’s and Children’s Health, Uppsala University, Akademiska sjukhuset, SE-751 85 Uppsala, Sweden; 4 Department of Women’s and Children’s Health, Karolinska Institutet, Tomtebodavägen 18A, SE-171 77 Stockholm, Sweden

**Keywords:** Attitudes, Embryo donation, Healthcare professionals, Paediatric, Reproductive medicine

## Abstract

**Background:**

The aim of this study was to investigate attitudes towards embryo donation and embryo donation families among professionals working in primary child healthcare, and their experiences of these families.

**Methods:**

A cross-sectional online survey was conducted in Sweden between April and November 2016. A total of 712 primary healthcare physicians, registered nurses and psychologists were approached to participate in this study. The study-specific questionnaire measured attitudes and experiences in the following four domains: legalisation and financing, the family and the child’s health, clinical experience of meeting families following embryo donation, and knowledge of embryo donation.

**Results:**

Of the 189 women and 18 men who completed the questionnaire (response rate 29%), relatively few (13%) had clinical experience of caring for families following embryo donation. Overall, 69% supported legalisation of embryo donation for infertile couples, and 54% agreed it should be publicly funded. The majority (88%) agreed the child should have the right to know the donors’ identity. Respondents did not believe that children conceived through embryo donation are as healthy as other children (50%), citing the risks of poor mental health (17%) and social stigmatization (18%). Approximately half reported low confidence in their own knowledge of embryo donation (47%) and wanted to know more (58%).

**Conclusions:**

These results indicate relatively large support among healthcare professionals in Sweden for the legalisation of embryo donation. In order to provide adequate healthcare to families following embryo donation, there is a need to develop educational resources to increase knowledge about the medical and psychosocial consequences of embryo donation among healthcare professionals working in primary healthcare.

## Background

Embryo donation is defined by the Ethics Committee of the American Society for Reproductive Medicine as a way for couples who have surplus cryopreserved embryos after in vitro fertilization (IVF) to dispose of these by donating them to individuals who need donor gametes or embryos [1]. Worldwide, there is a huge variety of laws and guidelines regarding embryo donation, which are shaped by various ethical issues and social and political structures [2]. Until recently, embryo donation was prohibited in Sweden because it was considered important for a child to be genetically related to at least one of its parents. However, it was recognized that genetic parentage does not necessarily imply good parenting, nor does it guarantee well-functioning families. Accordingly, embryo donation to couples and single women was permitted in January 2019. This decision was also in line with previous studies indicating support for legalisation among the Swedish general population [3], as well as among staff [4] and patients [5] at IVF clinics in Sweden.

Before the legislative change, Swedish women and couples travelled to other countries where embryo donation is permitted, such as Latvia, England or Spain. Consequently, children born following embryo donation are already present in the Swedish healthcare system. This raises the question if these children and families have specific needs related to the mode of conception. Only two studies were found that investigated children’s health and family adjustment following embryo donation, both based on a longitudinal study of 21 families. The results showed that the families were well-functioning and indicated no increased levels of emotional or behavioural problems in the children [6, 7]. However, compared to adoptive parents, embryo-donation parents exhibited higher levels of emotional over-involvement and reluctance to report negative feelings about parenting, and they were also more reluctant to disclose the child’s genetic origin [7].

Except in cases when gametes from oocyte and sperm donors are used to create an embryo, also called ‘double donation’, embryo donation results in a full genetic linkage between child and the donating couple. In addition, donors’ offspring are full siblings to the resulting child/children of their donated embryos. An interview study conducted in New Zealand with 22 embryo donors and 15 recipients found that both donors and recipients described the genetic link between donors and offspring ‘as bestowing immutable social ties’, and embryo donation was regarded as a way to build an extended family [8]. Thus, the legalisation of embryo donation raises important issues, such as the recipients’ lack of a genetic link to the child [9] and the potential for donors to regard the child as ‘theirs’ or as a sibling to their child [8]. Child-healthcare professionals may be in the position to provide information and support to families regarding these issues, but only if parents are open about the use of embryo donation to conceive.

Few studies have investigated healthcare professionals’ attitudes towards embryo donation and they have focused on details of the practice, such as accessibility for different groups and specific requirements for recipients [4, 10]. Research about attitudes in other areas of reproductive medicine has found that religion, profession [10], age and sex [11], personal experience of infertility [12] and clinical experience of the patient group [13] are associated with healthcare professionals’ attitudes towards assisted reproduction technology (ART).

Because knowledge about the health and functioning of children and families following embryo donation is limited, there is a risk of unsubstantiated opinions about these families taking hold in society. Earlier research in other areas indicates that societal attitudes do impact individuals’ experiences and may cause stigmatisation and exclusion, which in turn may negatively affect healthcare-seeking behaviour [14–16]. By investigating healthcare professionals’ perceptions and attitudes regarding embryo donation, we can identify areas in need of intervention and factors that may function as barriers in healthcare encounters. This is especially important as the number of families created through embryo donation is expected to grow. Therefore, the aim of the present study was to investigate attitudes towards embryo donation and embryo donation families among professionals working in primary child healthcare, as well as their experiences of these families. An additional aim was to identify background factors associated with displayed attitudes.

## Methods

### Context

Child-healthcare services in Sweden are included in the country’s publicly funded healthcare system and are free of charge. All children under the age of six receive regular check-ups at child-healthcare centres, typically on 12 occasions, of which 10 occur during the child’s first two years [17]. Paediatricians, nurses and psychologists work together at these centres to promote the best possible physical, mental and social health in children. This is achieved through different approaches, such as home visits, health monitoring, vaccinations and parental support. Open donation has been practiced in Sweden for more than 30 years and has until 2019 included treatment with either oocyte or sperm from a donor. The donor(s) and recipient(s) receive no information about each other, but the donor-conceived offspring has (at a mature age) the legal right to obtain identifying information about the donor(s). Thus, healthcare professionals at child-healthcare services in Sweden are likely aware of the national ‘open donation’ policy regarding oocyte and sperm donation.

### Sample and procedures

In 2016, between April and November, information about the study was sent to healthcare professionals at primary child-healthcare centres in four Swedish counties. The counties were both rural and urban with a total population of approximately 1.4 million people. From these counties 712 physicians, psychologists and registered nurses were invited by email to participate in this study. The email contained a letter of invitation outlining the study’s aim and procedure, as well as a link to the survey. The survey was completely anonymous in order to reduce possible social desirability when responding to the questions. Three reminders were sent to all possible participants to ensure that the request had been seen. The return of a completed questionnaire was regarded as giving informed consent.

### Measures

A study-specific survey was developed on the basis of clinical experience, earlier research and theory. In addition, items previously used to measure attitudes among healthcare professionals working in reproductive medicine were adapted and used [4, 18]. To ensure participants correctly understood what was meant by embryo donation, the survey started with an illustration and short description of the practice, as well as a statement noting that the practice, at that point in time, was not permitted in Sweden. The survey’s feasibility and face validity were evaluated by representatives from the target group, and minor changes and clarifications were made. The final survey consisted of 27 items.

Attitudes towards legalization and financing were measured with three items. The participants were asked to indicate the extent to which they agreed that embryo donation should be permitted in Sweden, if it should be publicly funded and if the child should have the right to know the identity of the donors. Responses were given using a five-point Likert scale ranging from ‘strongly disagree’ to ‘strongly agree’. For the purpose of analysis, the responses were dichotomised into ‘neutral/positive attitude’ and ‘negative attitude’.

Attitudes towards the family and the child’s health were measured with 14 items. The participants were asked to indicate the extent to which they agreed with different statements (e.g., ‘Children conceived through embryo donation display the same problems as adopted children’). Responses were given using a five-point Likert scale ranging from ‘strongly disagree’ to ‘strongly agree’. Responses were dichotomised into ‘disagree/neutral’ and ‘agree’.

The clinical experience of meeting families following embryo donation was measured with three items. Participants were asked to indicate whether they had met families with a child conceived through embryo donation at their clinic (yes or no). If they had, they were asked how they perceived these parents’ need for support in comparison to other parents’ needs. Responses were given using a five-point Likert scale ranging from ‘much lower’ to ‘much higher’. If respondents answered ‘somewhat higher’ or ‘much higher’, they were asked whether they had referred any of these families to specialist care for problems associated with the mode of conception (yes or no); then they were asked what kind of specialist care they recommended (open response format).

Knowledge of embryo donation was measured with two items. Participants’ confidence in their knowledge was queried with one statement: ‘I feel that I have sufficient knowledge about embryo donation and what it may imply for the child and family in order to provide adequate care’. Responses were given using five-point Likert scale ranging from ‘strongly agree’ to ‘strongly disagree’. The responses were dichotomised into ‘agree’ and ‘disagree’. Respondents were also asked if there was something about embryo donation they wanted to know more about (yes or no) and what specifically they wanted to know more about (open ended format).

Background variables included age, sex, profession and personal experiences of infertility in their own family or among friends. Sweden is generally a secular society, but there is ongoing debate regarding the introduction of a conscience clause. At the moment though, healthcare professionals do not have the right to refuse care based on personal beliefs or convictions [19]. Since having a strong belief, such as a moral conviction, has been shown to be associated with attitudes towards other aspects of reproduction [10, 12], the participants in the present study were also asked if they wanted a conscience clause to be introduced for healthcare professionals in Sweden.

### Data analysis

In order to test group differences in age, sex and education, one-way ANOVA and Chi square tests were used. The relationship between dependent variables (all displayed attitudes, *n* = 17) and independent variables was tested using univariable logistic regressions. Independent variables were chosen based on previous research and theory; these variables included age, sex, profession, experience of the patient group, personal experiences of infertility and the desire for a conscience clause [10, 12, 13, 18, 20]. Independent variables significantly correlated with any of the displayed attitudes (age, profession, personal experience of infertility and desire for a conscience clause) were entered into multiple logistic regression models. Nagelkerke’s R2 and the percentage of cases correctly classified were used to evaluate the models. One model (‘It is best for the child if the practice of how he/she was conceived is kept secret’) was discarded due to uneven distribution. As the analysis generated 16 models, the Bonferroni correction was used to adjust for multiple comparisons. Analyses were performed with IBM SPSS statistics 22 (IBM Corp. Westchester County, USA) and the significance-level was set at *p* < 0.05. Thematic content analysis [21] was used to analyse comments provided in an open response format, where words or phrases reflecting the same content were brought together to form categories.

## Results

A total of 712 healthcare professionals were invited to the study and 208 completed the questionnaire, yielding an overall response rate of 29.3%. Psychologists had the highest response rate at 55.9% (*n* = 19), followed by registered nurses with a response rate of 35.5% (*n* = 140) and physicians with a response rate of 17.3% (*n* = 49) (df 2, chi-square 38.947, *p* < 0.001). The mean age was 49.2 years (SD 10.45, range 27–68), with no difference between the professional groups (Table 1) or between sexes (men = 48.8 years and women = 49.2, t (192) = 0.153, *p* = 0.878). Almost half of the participants (47.6%) reported a personal experience of infertility in themselves, their family or among close friends. Almost a third (30.6%) of the physicians wanted a conscience clause for healthcare professionals to be introduced in Sweden, while only one in six of the registered nurses wanted one (16.4%) (df 1, chi-square 4.343, *p* = 0.037). None of the psychologists wanted a conscience clause to be introduced.

**Table 1:** Demographics of participants

Characteristics	Registered nurse (*n* = 140)	Physician (*n* = 49)	Psychologist (*n* = 19)	*P* ^b^
Age (mean, SD)	49.8 (10.32)	48.5 (10.30)	46.8 (11.78)	NS
	*n* (%)	*n* (%)	*n* (%)	
Sex^a^				
Female	139 (99.3)	32 (65.3)	18 (94.7)	< 0.001
Male	0	17 (34.7)	1 (5.3)	
Own experience of infertility				
Yes	59 (42.1)	27 (55.1)	13 (68.4)	0.048
No	81 (57.9)	22 (44.9)	6 (31.6)	
Wanting a conscience clause^a^				
Yes	23 (16.4)	15 (30.6)	0	0.009
No	115 (82.1)	34 (69.4)	19 (100)	

^a^Percentages do not sum to total due to missing values; ^b^ Between professional groups

### Attitudes towards legalisation and financing

The majority (69.2%) of respondents indicated they were positive or neutral towards embryo donation in Sweden (Table 2), and half were positive towards the public funding of embryo donation (53.8%). Most of the participants (87.7%) were positive or neutral towards the child having the right to obtain information about the identity of the embryo donors.

**Table 2:** Proportion of professionals who agree with, or were neutral about, the legalization and financing of embryo donation^a^

Attitudes^b^	Total	Registered nurse	Physician	Psychologist
	*n* (%)	*n* (%)	*n* (%)	*n* (%)
Embryo donation for infertile couples should be allowed	144 (69.2)	97 (78.9)	29 (69.0)	18 (94.7)
Embryo donation should be publicly funded	112 (53.8)	81 (65.9)	17 (41.5)	14 (73.7)
The child should have the right to know the embryo donors ID	157 (87.7)	105 (86.1)	35 (89.7)	17 (94.4)

^a^All participants did not answer all questions; ^b^Indicating 3 to 5 on a five-point Likert scale (neutral/agree/strongly agree)

The multivariable regression models showed that the desire for a conscience clause in Sweden was strongly associated with a negative attitude towards the legalisation (Odds Ration [OR] 5.40, confidence interval 95% [CI 95%] 2,11–13.85) and public funding (OR 3.30, CI 95% 1.28–8.53) of embryo donation. However, when adjusting for multiple comparisons, only the negative attitude towards the legalisation of embryo donation remained significant. Having a personal experience of infertility was associated with a positive or neutral attitude towards the legalisation (OR 2.76, CI 95% 1.16–6.60) and public funding (OR 3.12, CI 95% 1.52–6.38) of embryo donation, but only the attitude towards publicly funding embryo donation remained significant after adjustment for multiple comparisons. The professional and age groups had no independent impact on displayed attitudes.

### Attitudes towards the family and child’s health

About half of healthcare professionals (49.4%) agreed that children conceived through embryo donation are as healthy as other children (Table 3). About one in six participants believed that the child is at risk of poor mental health (16.6%) and social stigmatisation (18.3%), and one in five believed that children conceived through embryo donation display the same problems as adopted children (19.3%). However, few believed that the children are at risk of poor physical health (5.5%). While most agreed that it is important for parents to be honest with their child about the mode of conception (82.3%), there was some concern that the child’s knowledge about his/her conception could damage the relationship with parents (14.1%). There was also some concern that the child’s future contact with the donors could be harmful for the child and/or family (10.6%). Almost half of the respondents agreed that it would be positive for the child to have contact with the donors’ offspring, which would be his/her full sibling(s) (47.0%).

**Table 3:** Proportion of healthcare professionals agreeing ^a^ with statements about families through embryo donation

Attitudes^b^	Total	Registered nurse	Physician	Psychologist
	*n* (%)	*n* (%)	*n* (%)	*n* (%)
Parents are the ones who live with and take care of a child	166 (91.7)	114 (92.7)	37 (92.5)	15 (83.3)
A child must have a genetic link to at least one of the parents	11 (6.1)	8 (6.5)	3 (7.5)	0
Children conceived through embryo donation display the same problems as adopted children	35 (19.3)	24 (19.5)	10 (25.0)	1 (5.6)
The child is as healthy as other children	89 (49.4)	56 (45.5)	22 (56.4)	11 (61.1)
The child risks worse physical health	10 (5.5)	8 (6.5)	2 (5.0)	0
The child risks worse mental health	30 (16.6)	16 (13.0)	10 (25.0)	4 (22.2)
The parents are more involved in their children compared to those in other families	44 (24.7)	35 (28.7)	6 (15.8)	3 (16.7)
The child may experience a social stigma	33 (18.3)	21 (17.1)	9 (23.1)	3 (16.7)
When mature enough, it is good for the child to be able to know the identity of the donors	111 (61.7)	71 (58.2)	27 (67.5)	13 (72.2)
It is best for the child if the method of how he/she was conceived is kept secret throughout life	5 (2.8)	2 (1.6)	2 (5.0)	1 (5.6)
It is important that the parents are honest with the child with regard to how he/she was conceived	149 (82.3)	98 (79.7)	35 (87.5)	16 (88.9)
The child’s relationship with the parent could be damaged if he/she learns about the mode of conception	25 (14.1)	19 (15.8)	3 (7.7)	3 (16.7)
Contact with the donors (when mature enough) can be harmful for the offspring and/or the family	19 (10.6)	15 (12.3)	3 (7.7)	1 (5.6)
It is positive for the child (when mature enough) to be able to have contact with the donors’ offspring, which are the child’s full siblings	85 (47.0)	48 (39.0)	26 (65.0)	11 (61.1)

^a^Indicating 4 or 5 on a five-point Likert scale (Agree/Strongly agree); ^b^ All participants did not answer all questions

Multivariable regression models showed that the desire for a conscience clause was associated with respondents’ view that a child must have a genetic link to at least one of the parents (OR 6.78, CI 95% 1.63–28.23), that the child is at risk of poor mental health (OR 2.78, CI 95% 1.02–7.59) and that children born following embryo donation are subject to social stigmatisation (OR 4.16, CI 95% 1.59–10.90). Registered nurses less often agreed that it would be positive for the child to have contact with the donors’ offspring, compared to physicians and psychologists (39.0, 65.0 and 61.1% respectively, OR 0.35, CI 95% 0.16–0.75). Younger age was associated with the view that any contact with the donors could be harmful for the offspring and/or the family (OR 0.94, CI 95% 0.88–0.99). When adjusting for multiple comparisons, none of the attitudes remained significant. Having own experience of infertility had no independent impact on the attitudes displayed.

### Clinical experiences and perceived need for more knowledge

Relatively few respondents (24 nurses, three physicians and one psychologist) reported clinical experience of families with children conceived via embryo donation. Of these, five nurses and one psychologist (21.4%) perceived that these families had more need of support than other families, and two nurses had referred the child and/or family to specialist care; in one case to a counsellor for attachment problems and in the other case to a psychologist (reason not given).

In the total group of healthcare professionals, almost half (47.0%) reported insufficient knowledge to be able to provide adequate care for families who used embryo donation, with no difference evident between the professional groups. Further, more than half (57.5%) of respondents indicated a desire to learn more about embryo donation. Participants’ comments regarding their specific knowledge-needs were categorized into three areas.

In the area ‘The process of embryo donation and possible medical risks’, participants wanted to know how an embryo donation is performed and how the donors and receiving couple are evaluated. They also wanted to know more about possible medical risks in connection with the procedure, not only regarding treatment and during pregnancy, but also when it comes to the child’s future physical health. In the area ‘Psychosocial and psychological aspects of embryo donation’, participants wanted to know how embryo donation might impact the relationship between the child and its parents. This included wondering about early child-parent attachment. They also wanted knowledge about disclosure, including how disclosure may impact the child and family and in what way healthcare professionals can offer advice and support. In the last area, ‘Laws and regulations’, respondents wanted to know more about the legal aspects of embryo donation, both regarding the donors’ and recipients’ rights and security as well as those of the child.

## Discussion

The present study indicates that the majority of the healthcare professionals has a positive attitude towards embryo donation being permitted in Sweden and support the child’s right to learn about the embryo donors’ identity. Respondents expressed low confidence in their own knowledge about embryo donation and half believed children conceived by embryo donation were less healthy than other children. There was also concern that the child would risk poor mental health and social stigmatisation.

The finding that the majority of the respondents were positive or neutral towards the legalisation of embryo donation is in line with previous studies focusing on the general population, IVF couples and IVF staff in Sweden [3–5]. However, almost half of the respondents did not support publicly financed embryo donation. While the use of donated embryos is less medically complex and less expensive than gamete donation [1], embryo donation may involve additional costs, including counselling and consultation for both donors and recipients to ensure they understand the long-term implications for both themselves and their families [9, 22, 23]. It is acknowledged that the decision about the disposal of embryos is difficult. Feelings connected to relinquishing embryos include the absence of regret and the presence of altruistic emotions, relief and satisfaction, but also sadness, guilt and ambivalence [24]. A review study reported that these decisions are based on a range of factors, including the conceptualisation of embryos, such as their perceived moral status, meaning or symbolism, as well as perceived embryo quality [25]. However, independent of the meaning ascribed to embryos, donation entails a full genetic link between the donating couple and the resulting child, which may lead to more complex considerations than in the case of oocyte and sperm donation [26].

In the present study, the majority of respondents thought the child should have the right to learn the identity of the donors and held a positive view of the child learning about his or her genetic origin, which is in line with attitudes among healthcare professionals working in IVF clinics [4]. This result is not surprising as Sweden has practiced open donation for more than 30 years and the donor-conceived child’s right to information about his/her genetic origin is well-established. Even though it is strongly recommended that parents talk with their children about their donor conception [27], a meta-analytic review found that parents following embryo donation were less likely to disclose this information to the child compared to parents who used oocyte or sperm donation [28]. One possible explanation may lie in the complete lack of genetic connection to the parents. However, research has shown that fertility counsellors see as their main role to ensure that recipients fully understand the long-term implications of being parents through embryo donation [9, 23]. This role includes encouraging parents to reflect upon the meaning of genetic links and to consider the possible consequences of the child initiating contact with the donors and their full sibling(s).

In Sweden, fertility clinics only follow up on the medical outcomes of provided treatments. Any psychosocial support to donor-conceived families is regarded as the responsibility of child-healthcare services. In previous research focusing on Swedish families following gamete donation, significant groups of mothers (59%) and fathers (26%) expressed a need for information about how and when to talk to their children about donor conception [29]. In view of the complexity of parenthood following embryo donation, it is reasonable to believe these families will request professional support to manage disclosure and other issues, and that they will turn to healthcare professionals at child-healthcare centres. However, almost half of respondents in the present study reported insufficient knowledge in order to provide adequate care for embryo donation families, including how to advise and support parents about disclosure issues. This finding points to the importance of developing informational and educational resources to support both donor-conceived families and healthcare professionals working in primary care. Also, development of guidelines could be a way to ensure that parents receive the best advice about the process of disclosure.

About half of the respondents believed that it would be positive for the donor-conceived child, when sufficiently mature, to be able to have contact with the donors’ offspring, who would be their full sibling(s). This is in line with a review that found that many individuals conceived with donor oocytes or sperm wanted to know the identity of the donor in order to learn about their genetic origins, and many were also interested in contact with donor half-sibling(s) [30]. Also, a Finnish study reported that some embryo donors believed that contact between full siblings could be more important than contact with the donors (genetic parents), especially if the child does not have any siblings in his or her own family [31]. The importance of siblingship has also been reported by participants in the US Snowflakes© embryo adoption program, which is characterized by information-exchange and the possibility of ongoing contact between donors and recipients [32, 33]. These studies describe how embryo adoption creates new forms of siblingship, such as ‘batch siblings’ (i.e. embryos created at the same IVF cycle) and ‘genetic siblings carried by different mothers’. While some families established contact between genetic siblings early on, most decided to wait until the child was older. However, contact between donors and recipient families may be challenging, for example when embryo donors recognize recipients’ legal rights in relation to the resulting child but still regard the child as ‘theirs’ and as a sibling to their own children [8].

Embryo donation entails several psychosocial and ethical aspects, though knowledge about the outcomes for donors, recipients and the resulting children is currently limited. In the present study, one in five of the healthcare professionals believed that embryo donation-conceived children may face the same problems as adoptive children, who have been shown to experience increased levels of psychological morbidity, such as anxiety, depression and attention problems [34]. While both of these groups lack a genetic link to their parents, the conditions surrounding children conceived with embryo donation differ significantly from those of adoptive children who have often been exposed to negative experiences, such as institutionalisation and maltreatment. Our study also found that only half of the respondents believed that children conceived by embryo donation were as healthy as other children, and one in six thought that these children risk poor mental health and social stigmatisation. However, research has shown that families who used embryo donation function well and don’t experience increased levels of emotional or behavioural problems in comparison to adopted children or IVF children [6, 7]. This is in line with a review investigating long-term outcomes for families with children conceived through oocyte donation, which indicates that children and parents function well throughout childhood and into early adolescence [35]. Despite these reassuring findings, it is important to acknowledge that the research base is very limited, as embryo donation has only been available for a short time period in most countries. Further, even in jurisdictions where embryo donation has been available for a while, longitudinal and follow-up studies are rare because donations are often made anonymously.

In the present study, the desire for a conscience clause in Sweden was associated with a negative view of the legalisation and public funding of embryo donation, the belief that a child must have a genetic link to at least one parent and that children conceived through embryo donation are at risk for poor mental health. These findings are in line with earlier studies showing that the desire for a conscience clause [36] and having a strong religious belief [10] were associated with restrictive attitudes towards various aspects of reproductive medicine. If parents of embryo donation-conceived children turn to the primary healthcare system for support, it is important that the healthcare professionals act professionally and do not judge their choice to use embryo donation.

Personal experience of infertility emerged in this study as another factor associated with attitudes towards embryo donation. Respondents who had experienced infertility problems in their own family or among friends were more likely to have positive or neutral attitudes towards the legalisation and public funding of embryo donation. Similar results have also been shown in previous research [12]. This is not surprising, as personal experience may lead to increased empathy for the difficulties of involuntary childlessness, facilitating greater openness towards alternative forms of family-building.

### Methodological considerations

At the time of the present study, embryo donation was not permitted in Sweden and healthcare professionals working outside reproductive medicine may therefore have had limited knowledge of the practice. In order to ensure that respondents had a correct understanding of embryo donation, the survey included a short description and an illustration of the process. In addition, to make sure the survey items were clear and easy to understand, they were evaluated by one physician, one midwife and four registered nurses representing the target group. As the items measured opinions and attitudes and the expression of these opinions could be perceived as sensitive, study participation was completely anonymous. While the anonymous design reduced the risk of social desirability influencing responses, we cannot rule out the risk of selection bias. Also, the anonymous design made comparisons between responders and non-responders impossible. An invitation to participate in the study was sent to all individuals included in mailing lists at the selected primary child-healthcare centres. As the study was performed between April and November 2016, it is possible that some individuals on the mailing lists were not clinically active during the data collection period. While the response rate of the present study (29.3%) was equal to or higher than in earlier published survey-studies among healthcare professionals [37–40], the relatively high rate of non-responders does limit the external validity of the findings. Interestingly, the response rate was highest among psychologists (55.9%). It is possible that the study was more appealing to them because many items related to the psychological aspects of embryo donation. Relatively few respondents (13%) reported clinical experience of families with children conceived via embryo donation. However, parents may not necessarily have disclosed their use of embryo donation and, therefore, healthcare professionals may not have been aware of the child’s genetic origin. Also, relatively few men participated in the study and it is possible this had an impact on the result. The study reached enough power to make multivariable regression analysis possible, and the study was therefore able to identify factors associated with the participants’ attitudes towards embryo donation. The Bonferroni correction was used to adjust for multiple comparisons, which resulted in few independent variables that remained significant. Therefore, the results must be interpreted with caution.

## Conclusions

The present study indicates that primary child-healthcare professionals in Sweden are positive towards embryo donation being permitted in Sweden. However, they expressed concerns about the child’s health and risks of stigmatisation, and they expressed a desire to learn more about embryo donation in order to provide adequate care for this group of families. There is a need to develop educational resources in order to increase healthcare professionals’ awareness of the practice and implications of embryo donation, while also emphasising the limited research in this area. Since embryo donation entails specific challenges related to genetic linkages, child-healthcare professionals should be prepared to meet these families’ needs when it comes to disclosure and other issues related to this specific form of family-building.

## Data Availability

The datasets used during the current study are available from the corresponding author on reasonable request.

## Abbreviations

ART: Assisted Reproduction Technology
CI: Confidence Interval
IVF: In Vitro Fertilization
OR: Odds Ratio
